# Comparative sequence analysis of *Cyclospora cayetanensis* apicoplast genomes originating from diverse geographical regions

**DOI:** 10.1186/s13071-016-1896-4

**Published:** 2016-11-29

**Authors:** Hediye Nese Cinar, Yvonne Qvarnstrom, Yuping Wei-Pridgeon, Wen Li, Fernanda S. Nascimento, Michael J. Arrowood, Helen R. Murphy, AhYoung Jang, Eunje Kim, RaeYoung Kim, Alexandre da Silva, Gopal R. Gopinath

**Affiliations:** 1 Center for Food Safety and Applied Nutrition, U.S. Food and Drug Administration, Laurel, MD USA; 2 Division of Parasitic Diseases and Malaria, Center for Global Health, Centers for Disease Control and Prevention, Atlanta, GA USA; 3 Division of Foodborne, Waterborne, and Environmental Diseases, National Center for Emerging and Zoonotic Infectious Diseases, Centers for Disease Control and Prevention, Atlanta, GA USA

**Keywords:** *Cyclospora cayetanensis*, Apicoplast genome, Genomics, Next generation sequencing

## Abstract

**Background:**

*Cyclospora cayetanensis* is an emerging coccidian parasite that causes endemic and epidemic diarrheal disease called cyclosporiasis, and this infection is associated with consumption of contaminated produce or water in developed and developing regions. Food-borne outbreaks of cyclosporiasis have occurred almost every year in the USA since the 1990s. Investigations of these outbreaks are currently hampered due to lack of molecular epidemiological tools for trace back analysis. The apicoplast of *C. cayetanensis*, a relict non-photosynthetic plastid with an independent genome, provides an attractive target to discover sequence polymorphisms useful as genetic markers for detection and trace back analysis of the parasite. Distinct differences in the apicoplast genomes of *C. cayetanensis* could be useful in designing advanced molecular methods for rapid detection and, subtyping and geographical source attribution, which would aid outbreak investigations and surveillance studies.

**Methods:**

To obtain the genome sequence of the *C. cayetanensis* apicoplast, we sequenced the *C. cayetanensis* genomic DNA extracted from clinical stool samples, assembled and annotated a 34,146 bp-long circular sequence, and used this sequence as a reference genome in this study. We compared the genome and the predicted proteome to the data available from other apicomplexan parasites. To initialize the search for genetic markers, we mapped the raw sequence reads from an additional 11 distinct clinical stool samples originating from Nepal, New York, Texas, and Indonesia to the apicoplast reference genome.

**Results:**

We identified several high quality single nucleotide polymorphisms (SNPs) and small insertion/deletions spanning the apicoplast genome supported by extensive sequencing reads data, and a 30 bp sequence repeat at the terminal spacer region in a Nepalese sample. The predicted proteome consists of 29 core apicomplexan peptides found in most of the apicomplexans. Cluster analysis of these *C. cayetanensis* apicoplast genomes revealed a familiar pattern of tight grouping with *Eimeria* and *Toxoplasma*, separated from distant species such as *Plasmodium* and *Babesia*.

**Conclusions:**

SNPs and sequence repeats identified in this study may be useful as genetic markers for identification and differentiation of *C. cayetanensis* isolates found and could facilitate outbreak investigations.

**Electronic supplementary material:**

The online version of this article (doi:10.1186/s13071-016-1896-4) contains supplementary material, which is available to authorized users.

## Background

*Cyclospora cayetanensis* belongs to the phylum Apicomplexa, which is a large group of protists with phylogenetic ties to dinoflagellates and ciliates [1, 2]. Most apicomplexans are obligatory parasites causing several forms of human and animal diseases such as malaria (caused by *Plasmodium* spp.), toxoplasmosis (*Toxoplasma gondii*), coccidiosis in poultry (*Eimeria* spp.), babesiosis (*Babesia* spp.), theileriosis (*Theileria* spp.) and cryptosporidiosis (*Cryptosporidium* spp.) [3].

*Cyclospora cayetanensis* is a parasite recognized as a significant cause of diarrheal illness worldwide. Sporadic cases and outbreaks have been reported from many countries. When epidemiologic data are available most of the cases have been associated with the consumption of contaminated food and/or water [4–7]. Food-borne outbreaks of cyclosporiasis have been reported in the USA since the mid 1990’s [8] (http://www.cdc.gov/parasites/cyclosporiasis/outbreaks/index.html). Without molecular epidemiologic tools, it can be difficult to link cases to particular food vehicles and sources, thereby hampering the timely implementation of measures to control and prevent outbreaks. The development of molecular methods for the detection and characterization of *C. cayetanensis* isolates is therefore a priority for US public health agencies [9].

Apicomplexan parasites have an organelle called the apicoplast, a vestigial non-photosynthetic plastid originating from an ancient endosymbiotic algal ancestor [10–13]. Previous studies have shown that the apicoplast is involved in critical metabolic processes such as, heme and isoprenoid biosynthesis, fatty acid synthesis [11, 14–17], and is essential for growth in *Plasmodium falciparum* [18]. Because apicoplasts are vital to the survival of the parasites, they provide an attractive target for antiparasitic drugs [19, 20]. The sequence, gene content and map of various apicoplast genomes, including *C. cayatenensis* apicoplast genome, have been reported [21–25]. The apicoplast genomes of these parasites range 30 to 35 kb in size [3]. The structure and gene content of the apicoplast genomes are highly conserved; the genome of each apicomplexan species commonly encodes small subunit (SSU) and large subunit (LSU) rRNAs (rrs and rrl), three subunits of the bacteria-type RNA polymerase (rpoB, rpoC1, rpoC2), 16 ribosomal proteins, an EF-Tu, a ClpC-like protein and 24 tRNA species [3]. Most of the apicoplast genomes contain an inverted repeat (IR) consisting of *rrs*, *rrl*, and nine tRNA genes at both ends. Due to their non-recombining and co-inherited evolutionary nature, apicoplast and mitochondrial genomes have recently been used in the development of barcoding tools for tracking *Plasmodium* spp. [26–28].

Here we report the end-sequence curated and annotated complete reference genome for the *C. cayetanensis* apicoplast and present a proof of concept for using this reference to identify genomic markers for potential molecular epidemiology applications. Comparative analysis of sequence and gene organization of 11 *C. cayetanensis* apicoplast genomes originating from different geographical regions and the reference genome was performed. The results showed that the apicoplast genomes from *C. cayetanensis* strains are highly conserved with a few distinct polymorphisms. We identified 25 SNPs spanning the apicoplast genome, and a unique 30 bp- long repeat insertion sequence in a Nepalese sample. Phylogenetic comparisons of apicoplasts from different parasitic members of the Apicomplexa confirmed the existence of a conserved genomic structure and a common evolutionary history among these organisms. We identified a set of core proteins, conserved in many apicomplexan apicoplasts which potentially could be used for molecular typing and evolutionary studies. The SNPs and sequence repeats identified in this study could be used as genomic markers for source identification of outbreak strains of *C. cayetanensis* enabling molecular trace back analysis of outbreaks with high resolution.

## Methods

### *Cyclospora cayetanensis* samples

Some of the stool samples included in this study were originally submitted to CDC for confirmatory diagnosis of parasitic infections. Other stool samples containing *C. cayetanensis* oocysts were generously supplied by Professor Jeevan Sherchand, (Microbiology and Public Health Research Laboratory at Tribhuvan University Teaching Hospital in Kathmandu, Nepal), Ynes Ortega (The University of Georgia in Athens, Georgia, USA), Cathy Snider (Texas Department of State Health Services Laboratory), and staff at the Embassy of the United States in Jakarta, Indonesia. *Cyclospora cayetanensis* oocysts were purified from stool samples by a method similar to that developed for *Cryptosporidium* [29]. Briefly, *C. cayetanensis* oocysts were recovered from sieved fecal samples by differential sucrose gradient centrifugations (twice) and a cesium chloride (CsCl) gradient centrifugation. Sheather’s solution [500 g sucrose, 320 ml H_2_0 9 ml aqueous phenol (85%)] was used in 1:2, and 1:4 dilutions for sucrose gradient centrifugations. Gradients were prepared by pipetting 20 ml 1:2 Sheather’s solution and underlying it with 20 ml 1:2 Sheather’s solution, in 50 ml conical centrifuge tubes. Sieved fecal samples (10 ml) overlaid onto the gradient slowly. After centrifugation at 1000 g for 25 min. at 4 °C, oocysts were collected from 1:2 upper layer of the tube without disturbing the gradient. For cesium chloride gradient centrifugation; oocysts purified via sucrose gradient centrifugations were re-suspended in 0.5 ml saline, and carefully overlaid on 1 ml CsCl gradient solution (21.75 g CsCl- sp. gr. 1.15- in 103.25 ml dH_2_O) in a 1.7 ml siliconized micro centrifuge tube. Centrifugation was done at 16.000× *g* for 3 min. Oocysts were carefully collected from the layer between sample and CsCl fractions. Partially purified *C. cayetanensis* oocysts were counted using a hemocytometer and a Zeiss Axio Imager D1 microscope with an HBO mercury short arc lamp and a UV filter (350 nm excitation and 450 nm emission).

### DNA preparation and sequencing

Genomic DNA was isolated from *C. cayetanensis* oocyst preparations partially purified from clinical fecal samples, using the ZR Fecal DNA MiniPrep™ kit (Zymo Research, Irvine, CA, USA) following the manufacturer’s instructions with one modification. OmniLyse® cartridge (Claremont BioSolutions, Upland, CA, USA) was used to replace the bead beating step of the protocol provided by the ZR Fecal DNA MiniPrep™ kit. DNA concentration was measured with a Qubit 1.0 Fluorimeter using the Qubit dsDNA HS Assay Kit (Life Technologies, Grand Island, NY, USA). Whole genome sequencing (WGS) of the genomic DNA was performed on the Illumina MiSeq platform (Illumina, San Diego, CA, USA) using the Nextera XT, Nextera (Illumina), and Ovation (NuGEN, San Carlos, CA, USA) library preparation kits. Approximately 10 to 16 pmol of each library was paired-end sequenced on the MiSeq platform (Illumina).

### Bioinformatic analysis

The CLC Genomics Workbench toolkit (8.0) (Qiagen, Redwood City, CA, USA) was used for trimming the adaptor sequences from the whole genome sequencing (WGS) reads and subsequent genome assembly. Manual sequence curation was carried out in building the reference genome from a partial NGS assembly from NF1 sample and two contigs of HCNY WGS assembly (GenBank accession number LIGJ00000000). Bowtie2 (when custom databases were used for mapping on to selected genomes), and Geneious 9.0.5 (for mapping and visualization of the coverage). Mapped reads on Geneious 9.0.5 were used to generate consensus apicoplast sequences for manual curation. Blast analysis (NCBI reference) and Geneious 9.0.5 were used extensively to generate the final genome assembly by multiple alignments. An outline of the workflow used in sequenced analysis is given in Additional file 1: Figure S1.

A 300 bases long stretch of sequence (Additional file 2: Figure S2a) with the 30-bp unique insert was artificially created based on the end- and start- positions of a partial NF1 assembly. Read mappings and alignments were carried out using Geneious 9.0.5 and MEGA 7 suite as necessary. Specific reads from NF1 sample targeting this repeat region were manually curated to generate Fig. 5. Reads from other samples were mapped to this fragment resulting in a misalignment at the insert sequence to generate the illustration in Additional file 2: Figure S2b.

Initially, the reference apicoplast assembly was submitted to MAKER2 web annotation server (http://www.yandell-lab.org/software/mwas.html) for *de novo* annotation. RATT tool was used to transfer annotations mainly from *Eimeria tenella* (AY217738) [21] and also from a recently published *C. cayetanensis* apicoplast molecule (KP866208) [25]. Both versions of annotation of the reference genome were manually curated and corrected. An apicoplast core protein set was generated based on the presence of each of the reference genome assembly proteins available in the GenBank annotations for various species listed below. Specific alterations in the two *C. cayetanensis* consensus genomes were manually carried out in comparison with the reference genome using Geneious 9.0.5. Gaps (denoted by ‘N’) in the aligned genome assemblies were removed for phylogenetic analysis (resulting in Additional file 3). Neighbor Joining method was used as per the defaults on MEGA 6 and 7 [30] to build the phylogenetic tree with 12 *C. cayetanensis* apicoplast genomes (Additional file 4: Figure S4). CGViewer server located at http://stothard.afns.ualberta.ca/cgview_server/ was used to create visualization of in-built blast tool analysis. ProgressiveMauve [31] algorithm implemented in Geneious 9.0.5 as a plug-in was used for whole genome alignment and visualization for interspecies genome comparisons. The reference genome from this work was compared with *Babesia bovis* T2Bo (NC_011395), *Babesia microti* RI (LK028575), *Babesia orientalis* Wuhan (NC_028029), *Leucocytozoon caulleryi* (NC_022667), *Plasmodium chabaudi* (HF563595), *P. falciparum* HB3 (NC_017928), *Theileria parva-*Muguga (NC_007758), *E. tenella* (AY217738), *C. cayetanensis* HEN01 (KP866208), *T. gondii* (U87145), *Sarcocystis neurona* SN3 (contigs contig02351 and contig02350 from WGS assembly JAQE01002351) and *Neospora caninum* Liverpool (CADU01000154). To create *S. neurona* SN3 apicoplast, based on the description of the apicoplast in [32], the two contigs were aligned with the *T. gondii* genome and fused together to form a pseudomolecule for this work. GenBank annotations, wherever available, from these records were extracted and compared to generate a core apicoplast protein set.

## Results

### Assembly and annotation of the *C. cayetanensis* apicoplast reference genome

**Fig. 1:**
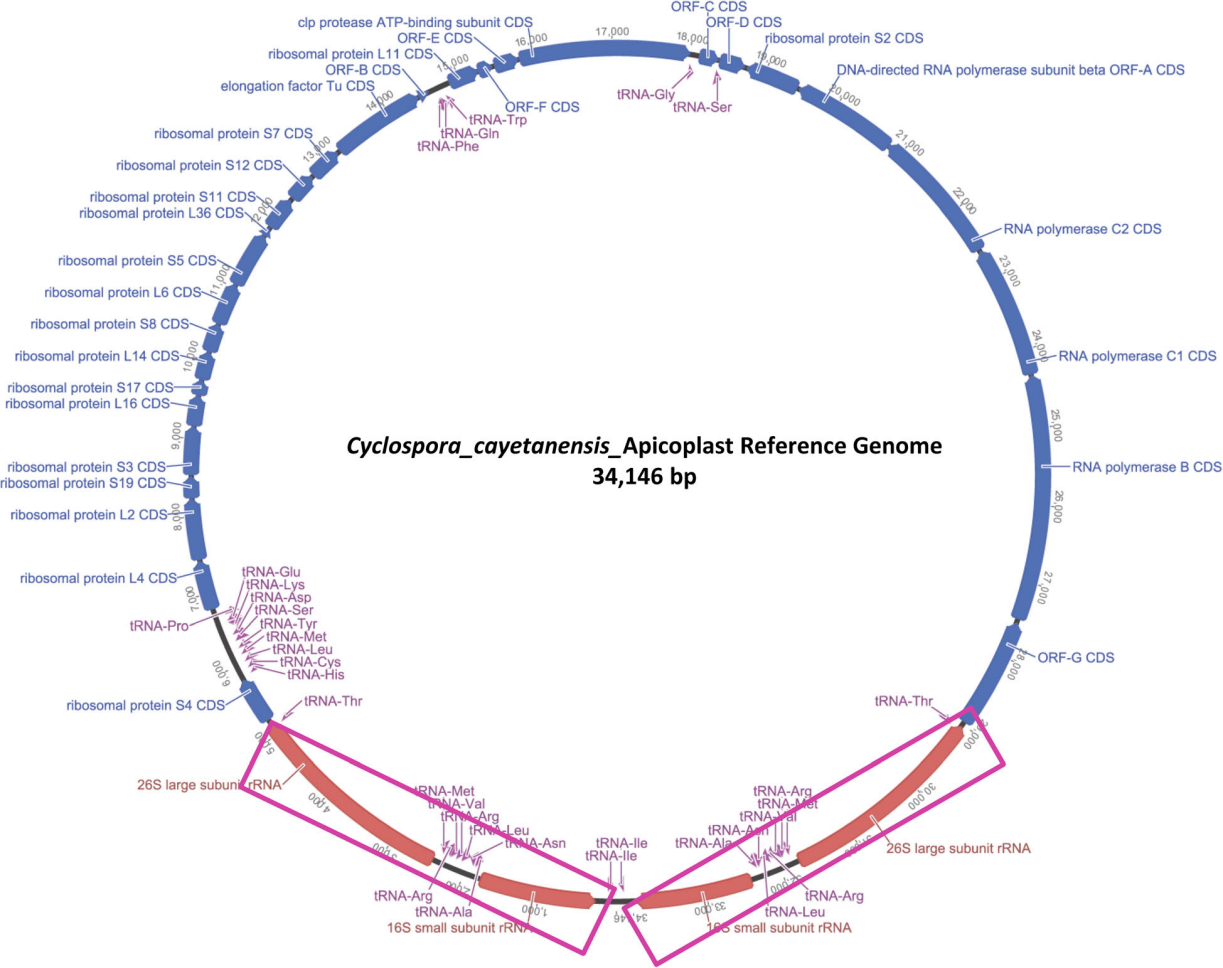
*Cyclospora cayetanensis* consensus apicoplast genome. The 34,146 nucleotide apicoplast genome was annotated using MAKER 2 server for *de novo* gene prediction; RATT tool [51] was used to transfer annotations from *E. tenella*. The predicted features were manually curated using comparison of the two annotation files. Sequences of the longest fragments of two terminal inverted repeat regions: 1–5147 and 28,999–34,146 bases, are shown in *purple boxes*

A local database of apicoplast genome sequences from *Eimeria* spp. and *C. cayetanensis* HEN01 was routinely used to collect apicoplast-specific reads from NGS datasets by Bowtie2 [33] mapping, and for identifying apicoplast contigs from genome assemblies by BLAST. An initial, gapped scaffold of the *C. cayetanensis* apicoplast genome was built using the NGS reads from a Nepalese sample NF1. By comparing 45 Mb HCNY *C. cayetanensis* draft genome assembly (LIGJ00000000) with our local database, two contigs (312 and 451) were identified. The main body of the apicoplast genome was coded by the 24 kb long contig 312 and the contig 451 constituted the approximately 5.5 kb fragment found as two inverted repeats in the apicoplast genome (Fig. 1). The NF1 scaffold was merged with the HCNY apicoplast contigs to create a consensus *C. cayetanensis* draft sequence assembly that was approximately 35 kb long. Approximately 25 million of WGS reads from samples NF1 and HCNY were mapped to this draft apicoplast genome using Bowtie2 and a mapping tool implemented in Geneious suite to collect reads targeting apicoplast sequences. Around one million of apicoplast-specific reads were used to refine the draft assembly of the artifacts arising from *de novo* assembly processes (such as insertion-deletions, extended end sequences, mis-assembly due to the ~5 kb repeats and random nucleotide polymorphisms). The *E. tenella* apicoplast genome (AY217738) was used to define the ends of the *C. cayetanensis* apicoplast assembly as it represented a typical apicoplast genome [21] from the closest species to *C. cayetanensis*. WGS reads from different sequencing runs using Nextera XT and Ovation libraries of NF1 and HCNY samples were used to verify the sequence at the nucleotide level. The manual curation of this draft sequence for error correction, confirmation of the end sequences, and resolution of the structure of the apicoplast genome by extensive reads-mapping, resulted in a circular, 34,146 bp long *C. cayatenensis* apicoplast genome (KX189066) which was used as the reference for intra-species and interspecies sequence comparisons in this paper (Fig. 1). In depth analysis with mapping of apicoplast reads from many samples confirmed the discrete difference in the length and terminal ends of this reference genome and the published genome from sample HEN01 (Additional file 5: Figure S5).

**Fig. 2:**
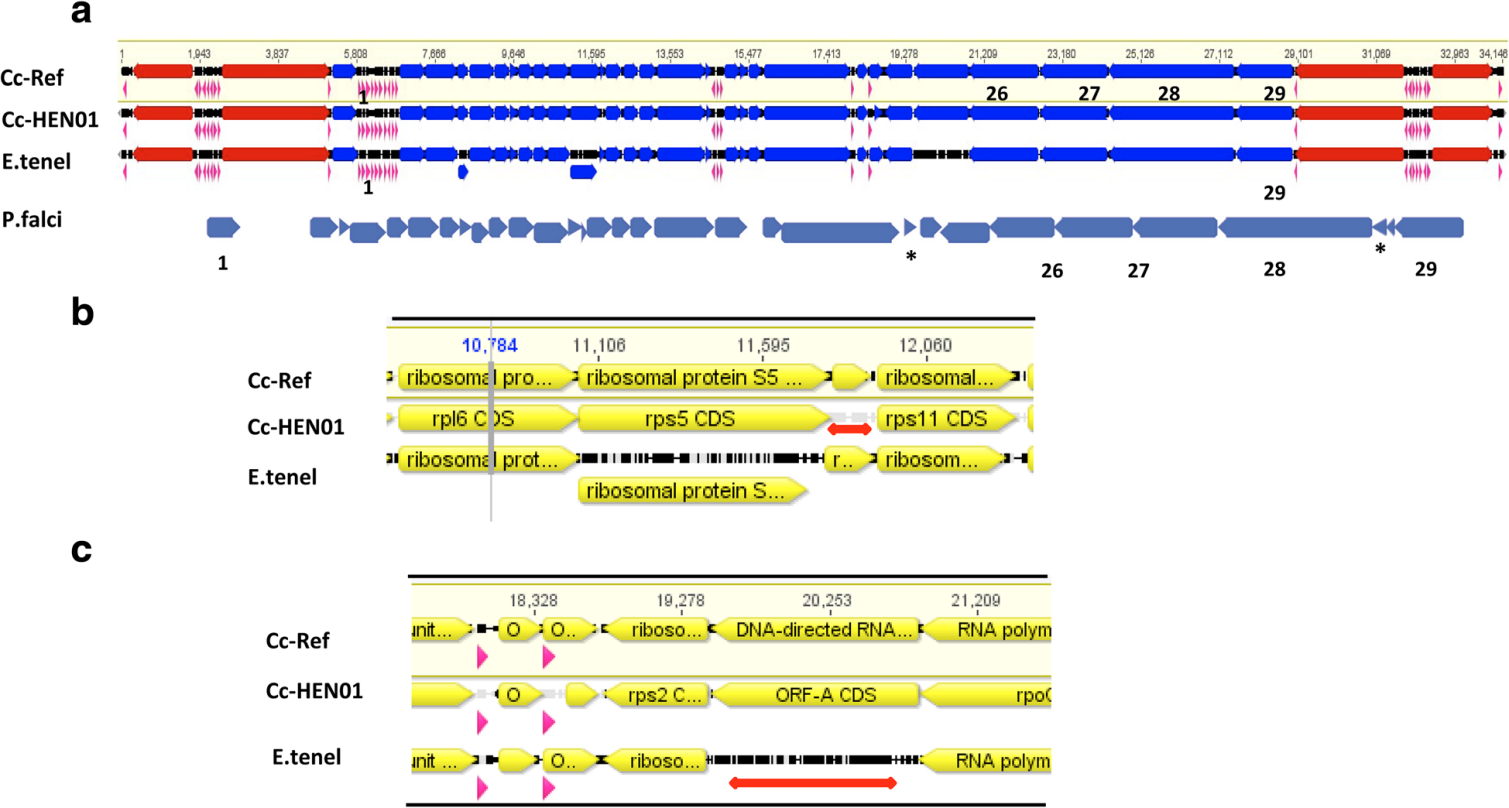
Complete apicoplast annotation and identification of core apicoplast proteins. **a** Draft annotations from *C. cayetanensis* reference genome (Cc-Ref) were aligned with KP866208 (Cc-HEN01 *C. cayetanensis* HEN01 strain), AY217738 (E.tenel: *Eimeria tenella*). The annotations were then manually curated to define gene boundaries (Table 1) and to correct any anomalies (**b** and **c**) in the assemblies. The final *C. cayetanensis* reference genome annotations were compared with LN999985 (P.falci: *Plasmodium falciparum* 3D7) and predicted proteins from other apicomplexan apicoplasts available in GenBank Twenty-nine core proteins present in most of the available apicoplast genomes were identified in the *C. cayetanensis* reference Table 1. Core apicoplast proteins 1–29 in *C. cayetanensis* (track Cc-ref) are illustrated above from left to right. In Tracks a-c, *arrows* in rRNA CDS are in *red*, tRNA in *purple* and proteins are in *blue*. In Track d, only the predicted proteome is shown in *dark blue*. **b** *Cyclospora* homolog of RPL36 (ribosomal protein L36) was included (track Cc-Ref) between RPS5 and RPS11 in the reference genome annotation. This partial peptide homologous to an *Eimeria* protein (track E.tenel) is not available (indicated by a *red line*) from the KP866208 genome annotation file in GenBank (track Cc-HEN01). **c** Based on the core apicoplast proteins identified in a wide variety of apicomplexan parasites, a homolog of ORF-A protein found in *C. cayetanensis* reference genome (track Cc-Ref) was predicted and confirmed to be present in *Eimeria* (track E.tenel). Currently this CDS is missing (indicated by a *red line*) from the annotations of *E. tenella* (AY217738) and other eimeriids. Track Cc-HEN01 represents the apicoplast coding regions of strain *C. cayetanensis* HEN01

Comparison of annotations of apicoplast genomes from *E. tenella* (AY217738), *C. cayetanensis* CHN HEN01 (KP866208), and *de novo* gene prediction of the new reference genome confirmed the presence of 29 protein coding genes, 33 tRNA and four rRNA genes (Fig. 2a and Table 1). The overall annotation of the *C. cayetanensis* apicoplast reference genome with respect to *E. tenella* and the *C. cayetanensis* HEN01 strain was highly comparable as illustrated in Fig. 2a with two exceptions: (i) A partial ribosomal protein L36 was missing from the HEN01 sequence (KP866208), but was retained in our reference genome annotation (Fig. 2b); (ii) A previously unannotated ORF-A gene was identified in the *E. tenella* GenBank record AY217738 (Fig. 2c). Predicted apicoplast proteins from other apicomplexan parasites listed earlier were compared to evaluate the *C. cayetanensis* reference genome annotations. Twenty-nine proteins found in the *C. cayetanensis* reference genome were found in most other apicoplasts and designated as the core apicoplast proteome in Apicomplexa (Fig. 2a, Track P.falci and Table 1, column 6). Core protein analysis facilitated identification of similar peptides with different names from different organisms. For example, peptides similar to SufB- or ycf24-domain containing protein ORF470 in *Plasmodium* spp. (LN999985 *P. falciparum* 3D7) are sometimes annotated as ORF-G in *C. cayetanensis* HEN01 and *E. tenella* AY217738 (peptide 29 in Fig. 2a). Furthermore, ORF-A is often reported to be missing in some apicomplexans [*Sarcocystis neurona* [32]; *T. gondii* (U87145); *E. tenella* (AY217738)] but we identified the protein in *E. tenella* (Fig. 2c), and in *T. gondii* assembly U87145, and in the *S. neurona* SN3 apicoplast pseudomolecule generated as part of this study (data not shown).

**Table 1:** Annotated genome of *Cyclospora cayetanensis* apicoplast

Name	Strand	Start	End	Length	Core Proteins	Name	Strand	Start	End	Length
ORF-G	-	27,539	28,969	1431	28	tRNA-Ile	+	34,061	34,132	72
RPOL B	-	24,324	27,488	3165	27	tRNA-Ala	+	32,287	32,359	73
RPOL C1	-	22,610	24,312	1703	26	tRNA-Asn	-	32,187	32,259	73
RPOL C2	-	20,830	22,590	1761	26	tRNA-Leu	+	32,102	32,181	80
ORF-A	-	19,469	20,827	1359	25	tRNA-Arg	+	32,025	32,098	74
RiboProtein S2	-	18,756	19,445	690	24	tRNA-Val	+	31,945	32,016	72
ORF-D	+	18,397	18,732	336	23	tRNA-Arg	-	31,855	31,927	73
ORF-C	+	18,134	18,391	258	22	tRNA-Met	-	31,771	31,844	74
clp protease	+	15,818	18,010	2193	21	tRNA-Thr	-	29,016	29,087	72
ORF-E	+	15,482	15,799	318	20	tRNA-Ser	+	18,391	18,475	85
ORF-F	+	15,267	15,464	198	19	tRNA-Gly	+	18,027	18,097	71
RiboProtein L11	+	14,874	15,263	390	18	tRNA-Trp	+	14,774	14,845	72
ORF-B	+	14,447	14,592	146	17	tRNA-Gln	+	14,692	14,763	72
elongation factor Tu	+	13,224	14,441	1218	16	tRNA-Phe	-	14,605	14,677	73
RiboProtein S7	+	12,765	13,190	426	15	tRNA-Pro	+	6684	6757	74
RiboProtein S12	+	12,369	12,734	366	14	tRNA-Glu	+	6593	6666	74
RiboProtein S11	+	11,912	12,331	420	13	tRNA-Lys	-	6491	6562	72
RiboProtein L36	+	11,776	11,892	117	12	tRNA-Asp	+	6404	6478	75
RiboProtein S5	+	11,055	11,774	720	11	tRNA-Ser	+	6312	6396	85
RiboProtein L6	+	10,503	11,051	549	10	tRNA-Tyr	+	6207	6291	85
RiboProtein S8	+	10,136	10,498	363	9	tRNA-Met	+	6125	6197	73
RiboProtein L14	+	9766	10,131	366	8	tRNA-Leu	+	6014	6100	87
RiboProtein S17	+	9555	9761	207	7	tRNA-Cys	+	5930	6000	71
RiboProtein L16	+	9165	9551	387	6	tRNA-His	+	5852	5925	74
RiboProtein S3	+	8533	9159	627	5	tRNA-Thr	+	5093	5164	72
RiboProtein S19	+	8246	8524	279	4	tRNA-Met	+	2336	2409	74
RiboProtein L2	+	7444	8241	798	3	tRNA-Arg	+	2253	2324	72
RiboProtein L4	+	6797	7435	639	2	tRNA-Val	-	2164	2235	72
RiboProtein S4	+	5211	5816	606	1	tRNA-Arg	-	2082	2154	73
16S SSU rRNA	+	32,403	33,905	1503		tRNA-Leu	-	1998	2078	81
26S LSU rRNA	-	29,033	31,746	2714		tRNA-Asn	+	1921	1993	73
26S LSU rRNA	+	2434	5147	2714		tRNA-Ala	-	1821	1895	75
16SSSU rRNA	-	275	1777	1503		tRNA-Ile	-	48	119	72

### *Cyclospora cayetanensis* apicoplast genome alignment with nine other apicomplexan species

**Fig. 3:**
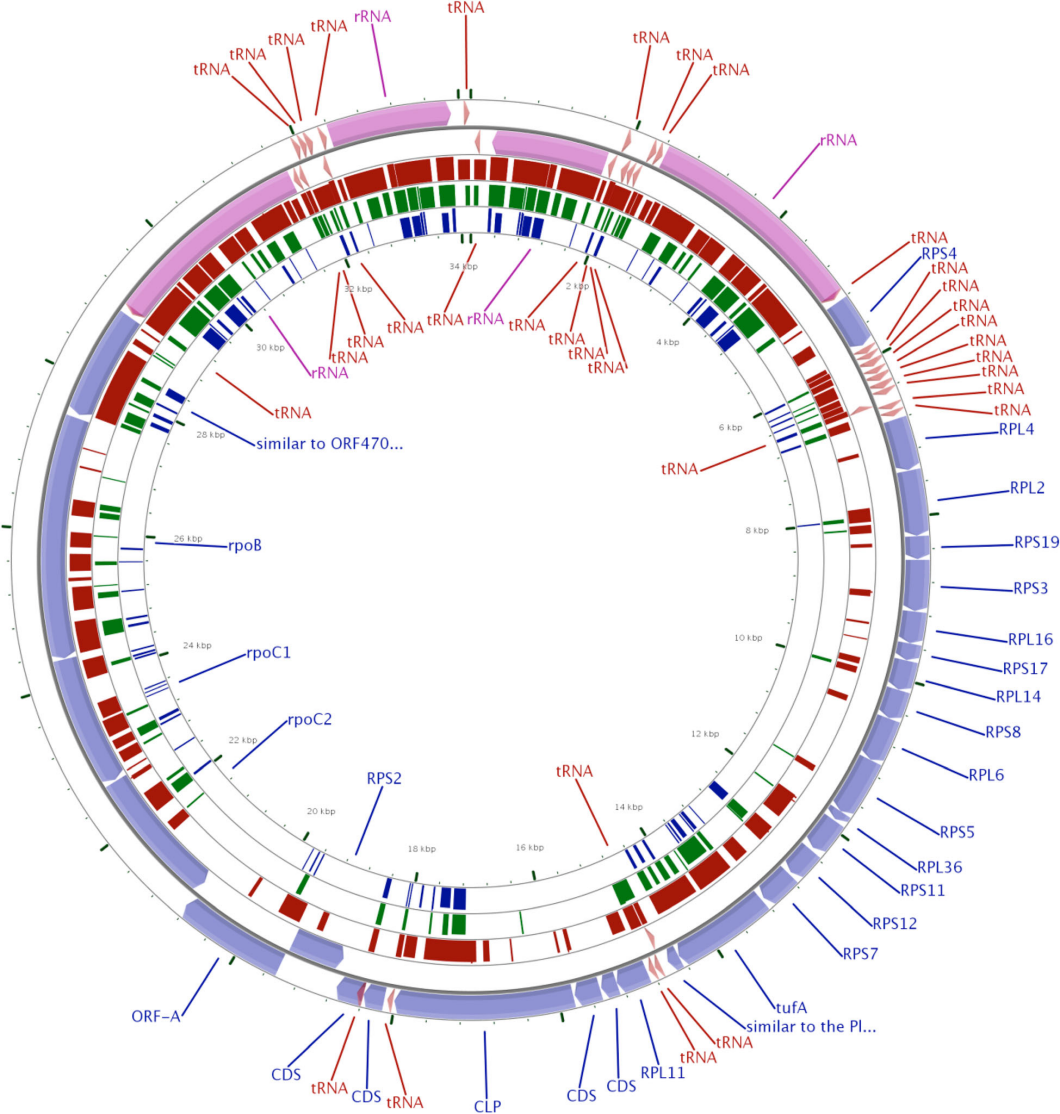
BLAST analysis of the *Cyclospora* apicoplast reference genome with other apicomplexans. Apicoplast genomes from an eimeriid, *E. tenella* and a sarcocystid, *T. gondii* (*brown* and *green bars*, respectively) and a plasmodiid, *P. falciparum* 3D7 (*violet*) are compared with the *C. cayetanensis* apicoplast (outer *circles* with *purple*, *blue* and *maroon* bands) using CGview software with its built-in Blast tool with the default e-value cutoff of 0.1. The thickness of the bands indicates sequence similarity. The eimeriid and sarcocystid apicoplasts have higher nucleotide similarity while the *Plasmodium* apicoplast is least similar of the three recapitulating evolutionary distance between these species. The predicted proteomes of these divergent apicomplexans contain mostly conserved core gene content (Fig. 2a)

**Fig. 4:**
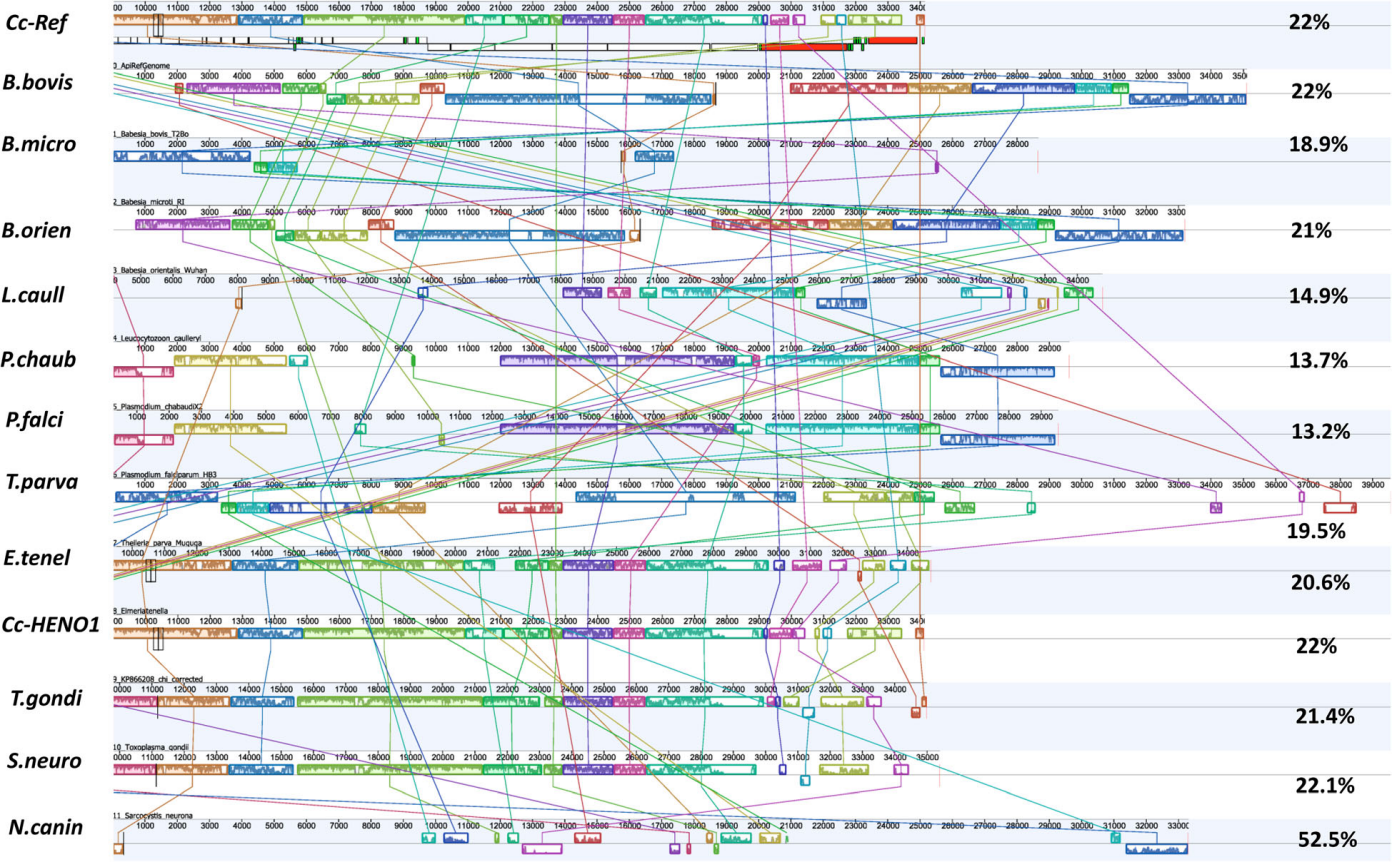
Whole genome alignment of *C. cayetanensis* apicoplast with 12 genomes from apicomplexan genera: *Babesia*, *Eimeria*, *Leucocytozoon*, *Neospora*, *Plasmodium*, *Toxoplasma*, *Theileria* and *Sarcocystis*. Whole genome alignment of (Track Cc-Ref) *C. cayetanensis* apicoplast reference genome with 12 assemblies from apicomplexans of the order Aconoidasida: (*B.bovis*) *Babesia bovis*; (*B.micro*) *B.microti*; (*B.orien*) *B. orientalis* Wuhan; (*L. caull*) *Leucocytozoon caulleyi*; (*P.chaub*) *Plasmodium chaubaudi chaubaudi*; (*P.falci*) *P. falciparum* HB3; (*T.parva*) *Theileria parva* and the order Conoidasida: (*E.tenel*) *E. tenella*; (*Cc-HEN01*) *C. cayatenensis* HEN01; (*T.gondi*) *T.gondii*; (*S.neuro*) *S. neurona*; and (*N.canin*) *N. caninum*. The GC content of the apicoplast genome from each species is displayed at the end of respective track. In addition, the apicoplast assemblies revealed re-arrangements in some species. Nucleotide divergence between members of Aconoidasida and Conoidasida is illustrated here

The genome structure of the *C. cayetanensis* apicoplast was compared with datasets from other apicomplexans in order to confirm the gene order and gene content. Whole genome comparison was carried out using NCBI Blast, Blast-feature built in CGViewer (Fig. 3) and Mauve implemented on the Geneious software suite (Fig. 4). The *C. cayetanensis* reference genome displayed 85% average nucleotide identity compared to *E. tenella* by NCBI Blast while this value decreased with *T. gondii* (72%) and *P. falciparum* 3D7 (68%). This significant local nucleotide divergence pattern from the Blast analysis was captured in the Fig. 3. When the multiple apicoplast genomes were aligned using Mauve algorithm, the varying lengths due to possible insertion and deletion of sequences are observed in Fig. 4. The GC content of the apicoplast genomes, marked on respective tracks in Fig. 4, ranged from 13.2 to 52.5% suggesting a diverse evolutionary history of these organisms. In spite of the species-level nucleotide differences, the apicoplast genomes in many parasitic apicomplexans appear to be globally comparable, as expected. For example, when the apicoplast genomes from *C. cayetanensis*, *Eimeria* and *Plasmodium* were compared, *Eimeria* showed higher sequence conservation to *C. cayetanensis* reference genome than to *Plasmodium* (Fig. 3). But the overall protein coding gene content appears not to have changed in these three species (Fig. 2a); all of the core proteins identified in *C. cayetanensis* were also present in *Plasmodium*, a relatively distant species. Similar pattern was observed when 12 apicoplast genomes from two distinct classes of Apicomplexa (Aconoidasida and Conoidasida) were compared in Mauve. Mauve analysis identified a varied number of blocks of sequence similarity which followed the line of evolutionary distance among distant genera like *Babesia*, *Plasmodium* and *Cyclospora*. In general, aconoidasidans like *Babesia* and *Theileria* had more in common with *Plasmodium* than with *Cyclospora* and other conoidasidans, and vice versa (Fig. 4) in terms of conserved sequence and structure. But the predicted apicoplast proteome of this diverse set of apicomplexans analyzed showed fewer deletions and protein coding gene acquisitions (data not shown; all comparisons based on GenBank annotations of the sequence files listed in the Methods). The determination of core apicoplast protein and comparison with other apicomplexans confirmed that the *C. cayetanensis* reference genome reported in this work is complete, and can be used for finding sequence variations.

### *De novo* assembly and genomic diversity of *C. cayetanensis* apicoplast genomes from geographically distinct samples

**Fig. 5:**
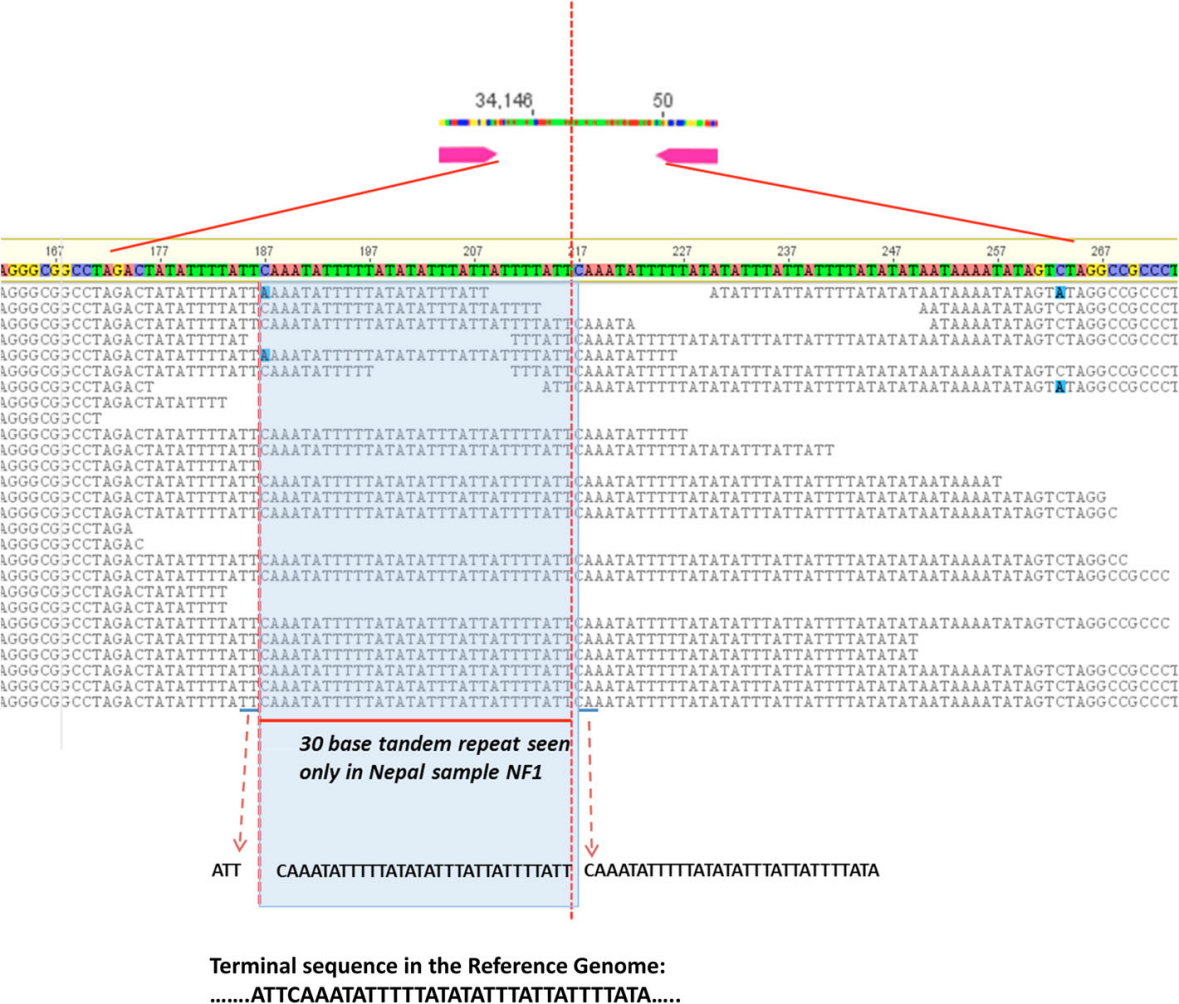
A 30 bp tandem repeat inserted into the terminal spacer sequences of NF1, a sample from Nepal. *Dashed vertical red line* represents the tail to head connection in the apicolast genome sequence. Multiple alignments of raw MiSeq reads are shown in *black*. Inserted bases are shown in the *blue box* with flanking end “ATT” and start “CAAA…..ATA” sequences. Typical terminal sequence stretch seen in Reference Genome is shown at the *bottom* of the illustration

Metadata details of the *C. cayetanensis* strains used for this work and the apicoplast genomes (annotated reference KX189066 and consensus assemblies) have been submitted to NCBI for immediate release under the community Bioproject ID: PRJNA316938. WGS datasets from *C. cayetanensis* samples from different geographical regions (Indonesia, Nepal, New York, Texas, Rhode Island, Virginia and Guatemala) and sequence from *C. cayetanensis* strain HENO1 (KP866208) were mapped to the reference genome as outlined in Additional file 1: Figure S1 and the resulting consensus assemblies (Additional file 3) were analyzed for structural genomic differences. A 60 bp long intergenic terminal region separating the two tRNA-Ile genes was found to be conserved in all but one of the samples*.* This consisted of a 14 bp repeat (34,133–34,146 and 33–46 base positions) adjacent to the terminal tRNA-Ile gene on each end (28 bp total). A 32 bp long terminal spacer region (1–32 base positions) flanks the repeat region near the start. However, the NGS reads from the FDA NF1 sample generated from 5 different libraries consistently failed to map to these terminal regions. Further analysis revealed an additional 30-base tandem duplicate of the terminal spacer inserted with a T/A base change at the end (Fig. 5). This terminal insertion was not identified in any other samples even after deep read-mapping analysis and was thus not included in the reference genome. When the reads were targeted to a 300 bp end to start sequence that included the unique insert from NF1 sample (Additional file 2: Figure S2a), rest of the geographical samples resulted in misalignment with ambiguous or missing base positions (Additional file 2: Figure 24b). Eleven complete apicoplast genomes from seven geographical regions validated by the depth of coverage and stringent quality assurance parameters were chosen for further analysis. When the apicoplast reference genome (KX189066) was compared with the published [25] *C. cayetanensis* HEN01 apicoplast genome (KP866208), we found more than two dozen positions with ambiguous bases, insertions and deletions, in the latter (Additional file 5: Figure S5). None of the anomalies in the original sequence of the KP866208 assembly were observed in any of the 11 WGS datasets analyzed in this work. It was necessary to re-order the start of the KP866208 to reflect the validated end-start junction site of the newly derived reference genome. Thirty three bases from the tail end of KP866208 GenBank sequence was moved to the beginning. In addition, 19 deletions and 10 replacements resulting in a net decrease of 9 bases were carried out to reconcile the KP866208 with our reference genome. The ambiguous bases in the corrected KP866208 were replaced with ‘N’ (Additional file 3).

**Fig. 6:**
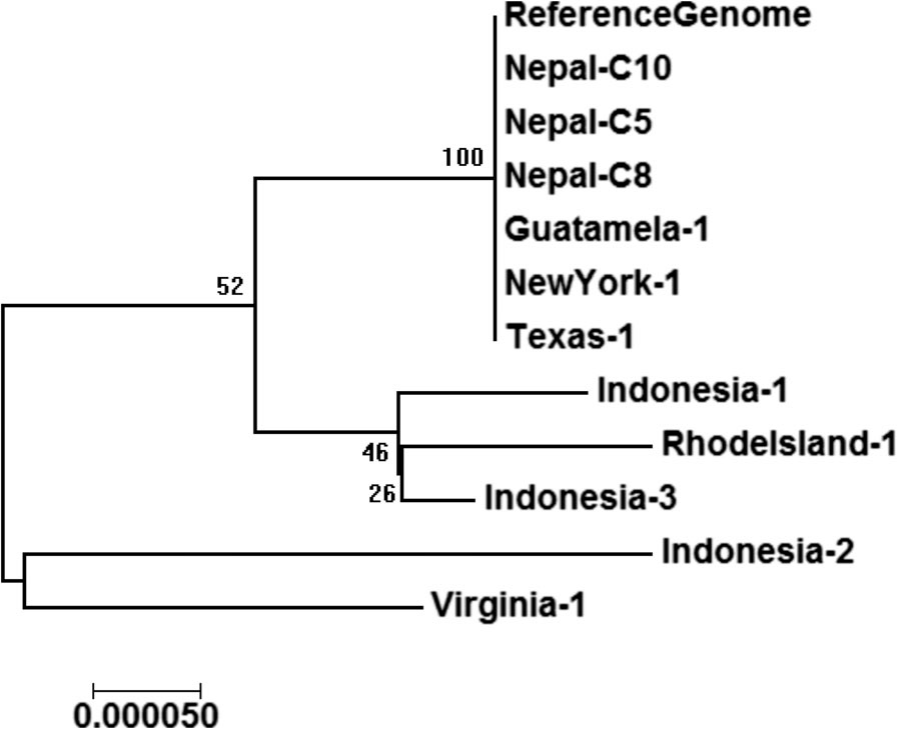
Phylogenetic analysis of 11 *C. cayetanensis* apicoplast genomes from seven geographical regions compared with the reference genome. The percentage of replicate trees generated using Neighbor-Joining method in which the associated taxa clustered together in the bootstrap test (500 replicates) is shown next to the branches. Evolutionary analyses were conducted in MEGA7 suite [30]

Eleven apicoplast consensus genomes derived from WGS reads (Additional file 3) were aligned against the 34,146 bp long reference genome to determine the presence of SNPs and other genomic variations or differences. Single samples each from Rhode Island, Texas, and Virginia and three samples from Indonesia showed distinct polymorphisms and indels spanning their whole genomes. Indonesia-2 and Virginia-1 samples exhibit tetrameric polymorphic repeats of A and T (Table 2). Various combinations of SNPs located in 21 positions spanning these apicoplast genomes could distinguish samples from Virginia, Indonesia and Rhode Island from those which originated from Texas, Nepal, Guatemala and New York (Table 2). Based on this ungapped alignment (Additional file 3 with 12 genomes without any gaps), a phylogenetic tree was built (Fig. 6). As expected from their alignments with the reference genome, the identical genomes of samples from Texas, New York, Nepal and Guatemala clustered together. Interestingly the three Indonesian samples appeared in separate clades indicating more extensive diversity underlying these samples from the same geographical location.

**Table 2:** Nucleotide variations in the apicoplasts from geographical samples compared to the *C. cayetanensis* apicoplast reference genome

Base position	Ref Genome	Rhodeisland-1	Indonesia-1	Indonesia-2	Indonesia-3	Virginia-1
1	C	T	T	T	T	C
533	T	T	T	C	T	T
557	TTTT	AAAA	TTTT	TTTT	TTTT	AAAA
1898	A	T	T	T	T	A
2242	T	T	A	A	T	T
6002	C	C	C	C	A	C
6110	T	A	A	A	A	T
7047	C	C	C	C	C	T
7373	G	T	G	T	G	G
7374	A	C	A	C	A	A
7780	T	T	GGAT	T	T	T
10,623	C	A	C	C	C	C
12,759	T	DEL	T	T	T	T
12,760	A	DEL	A	A	A	A
14,600	A	A	DEL	A	A	A
15,493	A	T	T	T	T	A
18,625	T	A	A	A	A	T
18,629	T	C	T	T	T	T
20,515	G	G	G	G	G	A
20,555	T	T	T	T	T	G
26,122	T	T	G	T	T	G
31,938	A	A	T	T	T	A
32,282	T	A	A	A	A	T
33,620	AAAA	AAAA	AAAA	TTTT	AAAA	TTTT
33,647	A	A	A	G	A	A

## Discussion

We found distinct differences between the *C. cayetanensis* apicoplast reference genome (KX189066) reported in this work and the previously reported *C. cayetanensis* genome assembly KP8666208 [25]. Although the annotation of both assemblies was highly similar (Figs.1 and 2a, Table 1), we found more than two dozen positions with ambiguous bases, insertions and deletions (Additional file 5: Figure S5). Interestingly none of these sequence variations were shared with any of the other 11 genomes we assembled in this study and so we removed the KP8666208 genome assembly from our clustering analysis but included it for other comparisons. The 30-bp tandem duplicated insertion in the spacer region of FDA-NF1 sample (Fig. 5) was unique to this particular sample (comparative genomic analysis details provided in Additional file 2: Figure S2). Tandem repeat structures are common in chloroplast sequences in both coding and non-coding regions and it would be interesting to capture multimeric nucleotide variations if any are found in *C. cayetanensis* apicoplasts. Due to its polymorphic nature and co-dominant mode of inheritance, these repeat stretches have been used as DNA markers for population genetics studies [34–37]. The biological significance of this finding in *C. cayetanensis* remains unknown.

Genetic variation in apicomplexans such as *P. falciparum* has been shown to reflect geographic distance, and population dynamics [26]. Organelle genomes are particularly informative in tracing patterns of population dynamics due to their non-recombining nature. Mitochondrial and plastid sequences have been used to search for the origins of humans [38], grapevines [39], and have also served as DNA barcodes for plants and animals [40]. We sequenced and analyzed 11 complete *C. cayetanensis* apicoplast genomes from seven geographical locations of sample collection to determine whether SNPs and other sequence signatures may be geographically informative. SNPs and indels identified in this study span all of the *C. cayetanensis* apicoplast genome and provide a higher resolution with distinct distinguishing power when compared to single gene or repeat sequence based efforts reported in the past [7, 41, 42]. The comparative genomic analyses have demonstrated the genomic diversity of *C. cayetanensis* apicoplasts among the sub-groups of samples used. The approach used in this study resulting in observations of inherent genomic variations in the geographical isolates may be used to develop a strategy to discern a more comprehensive survey of genetic variations from worldwide samples in the future. Apicomplexans like *Plasmodium* have been reported to contain a few hundred SNPs in their apicoplast genomes when larger populations of samples from many geographical areas were analyzed [26]. It is evident from Fig. 6 that the 34 kb long *C.cayetanensis* apicoplast genome may harbor more variations than observed in this analysis with a limited number of samples (*n* = 11) from 7 geographical locations. Moreover, *Cyclospora* is found to be common in tropical and subtropical regions but spread to countries importing the foods contaminated by the parasites [https://www.cdc.gov/parasites/cyclosporiasis/epi.html]. Although the 11 samples clustered into 5 distinct groups based on approximately 25 SNP positions, the travel history of the patients and sources of contamination leading to the illness from each of the US sample collection locations could not be easily verified. Thus, we cannot determine with certainty that the sequence variations are geographically specific in those US patient samples that clustered together (Fig. 6). This highlights the necessity of collecting critical metadata of the source of sample collection to capture important information on the occurrence of the parasites in different regions. Increased number of samples from various geographical regions where *C. cayetanensis* is detected in foods, water or in patients coupled with clear epidemiological data would provide a better perspective of the genomic diversity. In order to encourage the *Cyclospora* research community to build a resource of apicoplast genomes for identifying strain-level variations from different parts of the world, we have created a “Bioproject” in NCBI titled “*Cyclospora cayetanensis* Geo-Genomic Profiling using Apicoplast Genomes from different Geographic Areas” (http://www.ncbi.nlm.nih.gov/bioproject/PRJNA316938) for submission of sequence data, both raw (“SRA”) and assembled (“Assembly”), and sample metadata (“BioSample”). Global researchers of *Cyclospora* spp. can submit their datasets to this open BioProject with assistance from the authors (GG and AD) of this work.

Whole genome assemblies from WGS reads are prone to sequence errors due to read quality and assembly processes. The apicoplast genomes are marked by a pair of 5 kb low GC content terminal repeats constituting almost 30% of the total length. The problem of missed, wrongly annotated or fragmented assemblies (present in different contigs of a WGS assembly) is characteristic of genome assemblies from NGS reads [43]. For these reasons, we evaluated the quality of our reference assembly, by comparing the predicted proteome of related parasites including *Cyclospora*, and *Babesia* among other apicomplexans, and defined a set of core apicoplast proteins found in most of the apicomplexans. Across the apicomplexan species we tested, the gene content seemed to be mostly preserved with only minor changes. In addition, the annotated core apicoplast proteins (Table 1; column 6) identified based on many apicomplexan parasites allowed a comparative genomics approach to correct a few nomenclature or mis-identification issues in published apicoplast genomes. Apicoplasts from different apicomplexans have been previously noted to contain a set of proteins with similar essential biochemical functions [44]. The analytical approaches to study the phylogenetics of apicomplexan parasites have been expanding to include more loci from mitochondria, apicoplasts and chromosomal genomes. Recently, a combination of mitochondrial, apicoplastic and chromosomal genes were found [45] to be sufficient to infer the evolutionary relationships among Eimeriid coccidians. Current work on the *C. cayetanensis* reference genome expands the repertoire of target sequences for extended phylogenetic analysis of apicomplexans. The genome alignment in Fig. 4 includes Aconoidasidans, (like *Plasmodium* and *Babesia*) and Conoidasidans (like *Eimeria*, *Toxoplasma* and *Cyclospora*) which represent differences in life cycle, host, genome size, GC content of apicoplast genomes and taxonomic positions. The range of GC content among apicomplexans from 13% (in Plasmodium), 26% (in *C. cayetanensis)* to 52.5% (in *Neospora*) is reflected in the gaps in the Mauve multiple alignment (Fig. 4). The uniformity seen in the genomes across multiple species based on available sequences confirms a suggested stable evolutionary state of plastid evolution from a common ancestor prior to species diversification [46]. It supports for the functional importance of apicoplasts as noted in other earlier reports [11, 47].

*Cyclospora cayetanensis* has emerged as a cause of diarrheal illness in different countries where cases of infections were not known. A large number of cases have been reported from developed and developing countries in the form of seemingly sporadic cases or outbreaks associated with food- or waterborne transmission. Consumption of imported produce has been associated with U.S. outbreaks of cyclosporiasis. Thus, it is reasonable to believe that the globalization of the food supply has been a key element for the spread of *C. cayetanensis* infections. There have been relatively few U.S. outbreaks of cyclosporiasis for which a food vehicle was definitively identified [48]. It is not yet clear about the potential food vehicles that can be associated with reported outbreaks during any given year. During the 2013 Texas cyclosporiasis outbreak investigation it was clear that some outbreak cases were associated with consumption of cilantro imported from the state of Puebla in Mexico. However, no definitive food vehicle was implicated for the majority of the cases reported during the outbreaks [9]. The investigations that took place in 2013 illustrated essential scientific gaps that needed be closed in order to streamline outbreak investigations; i.e. (i) absence of more sensitive and specific diagnostic methods that can improve case detection; (ii) lack of molecular epidemiology methods to link cases to each other or to particular food items; and (iii) the absence of practical tools to detect the organism in food and potential sources of contamination in the environment.

## Conclusions

This study focused on closing the molecular epidemiology gap to help link clinical cases to each other, and to particular food items. The availability of genomic data and associated sample metadata from across the world should accelerate the profiling of *C. cayetanensis* isolates or even species of *Cyclospora* from diverse sources samples, e.g. zoonotic samples [49, 50] possibly leading to development of molecular tools for identification and source-tracking. The WGS-based reference genome reported in this work was completed by high quality, in depth read-mapping and comparative genomics. In the process, we have developed a framework to perform in-depth intra- and inter-species comparisons of apicoplast genomes to study the evolutionary relationship of apicomplexan parasites, and to identify specific variations in *C. cayetanensis* strains.

## Additional files

Additional file 1: Figure S1. Workflow used in this study for variant detection in apicoplast sequences. (TIF 172 kb)
Additional file 2: Figure S2. 300 bp end to start sequence analysis. A) 300 bp end to start sequence with a unique insert. B) Alignment of corrected and assembled reads from NF1 samples and 10 geographical samples with the 300 bp end sequence from NF1 assembly (sample #1). Except for the reads from NFI (sample #2), rest of the samples (samples #3-#12) do not map to this fragment containing the unique insert sequence. (TIF 8706 kb)
Additional file 3: 33,598 bases long ungapped apicoplast genome sequences for building the tree in Fig. 5. KP866208_corrected was not included in this analysis. The sequences were first aligned on MEGA7 and bases positions with ‘N’ were removed from all strains to create an ungapped sequence file. MEGA 7 suite was used for this analysis as described. (FASTA 409 kb)
Additional file 4: Figure S4. 34,146 bp apicoplast reference genome and consensus sequences from 11 geographical strains used in the multiple alignment to identify genomic changes and clustering analysis. KP866208 was not used in the clustering analysis. Indonesia-1 apicoplast is 34,148 long with a missing base (filled in as N) in 14,602 and a ‘GGA’ insertion in 7780 base positions. Virginia-1 consensus sequence is 33,625 bases long reflecting coverage gaps. Rhodeisland-1 sequence is 34,132 bases long with gaps at 12,579-80 and 15,372-383. All gaps were filled with N for multiple-alignment and clustering analysis. The base positions are for the Reference Genome. (TIF 105 kb)
Additional file 5: Figure S5. KP866208 sequence from GenBank was aligned to the reference genome KX189066 and reconciled by making the following corrections. Deletions and ambiguous (“AMBI”) bases were replaced with N. N/A indicates ‘relevant information not available’. The corrected sequence is 34,146 bp long, similar to the reference sequence. Bars in the KP866208 genome represent SNPs or indels in comparison to the reference genome. Starting length: 34,155 bases. There were 10 insertions and 19 deletions [34,155 + 10 − 19 = 34,146 bp (the length of the reference genome)]. (TIF 3729 kb)

## Abbreviations

CDC: Center for Disease Control and Prevention
CsCl: Cesium chloride
FDA: Food and Drug Administration
indel: Insertion/deletion
IR: Inverted repeat
kb: Kilobase
LSU: Large subunit
Mb: Megabase
rrl: Large subunit ribosomal RNA
rRNA: Ribosomal RNA
rrs: Small subunit ribosomal RNA
SNP: Single nucleotide polymorphism
SSU: Small subunit
tRNA: Transfer RNA
WGS: Whole genome sequencing
